# Inhibitory Effects of 3-Cyclopropylmethoxy-4-(difluoromethoxy) Benzoic Acid on TGF-β1-Induced Epithelial–Mesenchymal Transformation of In Vitro and Bleomycin-Induced Pulmonary Fibrosis In Vivo

**DOI:** 10.3390/ijms24076172

**Published:** 2023-03-24

**Authors:** Tianxiao Sun, Haihua Li, Yan Zhang, Guixin Xiong, Yuerun Liang, Fang Lu, Rong Zheng, Qi Zou, Jiejie Hao

**Affiliations:** 1 Key Laboratory of Marine Drugs, Ministry of Education, School of Medicine and Pharmacy, Ocean University of China, Qingdao 266003, China; 2 Laboratory for Marine Drugs and Bioproducts, Pilot National Laboratory for Marine Science and Technology (Qingdao), Qingdao 266237, China

**Keywords:** IPF, A549, EMT, TGF-β1/Smad, ECM

## Abstract

Idiopathic pulmonary fibrosis (IPF) is a progressive lung disease characterized by lung inflammation and excessive deposition of extracellular matrix components. Transforming growth factor-β1 (TGF-β1) induced epithelial–mesenchymal transformation of type 2 lung epithelial cells leads to excessive extracellular matrix deposition, which plays an important role in fibrosis. Our objective was to evaluate the effects of 3-cyclopropylmethoxy-4-(difluoromethoxy) benzoic acid (DGM) on pulmonary fibrosis and aimed to determine whether EMT plays a key role in the pathogenesis of pulmonary fibrosis and whether EMT can be used as a therapeutic target for DGM therapy to reduce IPF. Firstly, stimulation of in vitro cultured A549 cells to construct EMTs with TGF-β1. DGM treatment inhibited the expression of proteins such as α-SMA, vimentin, and collagen Ⅰ and increased the expression of E-cadherin. Accordingly, Smad2/3 phosphorylation levels were significantly reduced by DGM treatment. Secondly, models of tracheal instillation of bleomycin and DGM were used to treat rats to demonstrate their therapeutic effects, such as improving lung function, reducing lung inflammation and fibrosis, reducing collagen deposition, and reducing the expression of E-cadherin. In conclusion, DGM attenuates TGF-β1-induced EMT in A549 cells and bleomycin-induced pulmonary fibrosis in rats.

## 1. Introduction

Idiopathic pulmonary fibrosis (IPF) is a chronic and progressive interstitial lung disease that progresses to loss of lung function [1]. IPF is characterized by structural remodeling of lung tissue, inflammatory responses in the interstitium of the lung, destruction of the alveolar structure, and massive deposition of the extracellular matrix (ECM) components in the interstitium and in the basement membrane, resulting in progressive destruction of the lung structure and irreversible loss of lung function [2,3,4]. The pathogenesis of IPF involves several risk factors, including environmental exposure, smoking, chronic viral infections, and certain co-morbidities, among which genetic risk factors are the most critical [5]. IPF has a median survival time of 2 to 4 years [6]. The incidence of IPF in North America and Europe is higher than in South America and East Asia, and is more prevalent among older patients [4,7]. Nintedanib and pirfenidone (PFD), which were approved by the United States Food and Drug Administration in 2014 for the treatment of IPF, can restore vital capacity of the lung, but are not associated with a reduction in mortality [8]. With the high price of treatment drugs and the compromised quality of life, IPF patients are under great financial and psychological burden.

Many studies have shown that the pathogenesis of IPF is closely related to several growth factors, cytokines, and chemokines involved in regulating coagulation, angiogenesis, inflammation, and repair responses. These factors affect biological pathways that guide cellular responses in IPF, including activation of alveolar epithelial cells, fibroblast foci formation, and abnormal extracellular matrix (ECM) deposition leading to lung parenchymal remodeling [9].

Currently, the etiology of IPF is unknown, but the recently popular hypothesis is that the fibrotic cascade begins with defective alveolar epithelial II (ATII) cells that cannot transdifferentiate into more widely distributed alveolar epithelial (ATI) cells, thereby regenerating the alveolar epithelium after injury or during inversion [10]. In the context of the pathogenesis of IPF, the AE2 cells have been assigned a central role [11]. Epithelial–mesenchymal transformation (EMT) has made great progress in understanding the pathogenesis of pulmonary fibrosis. EMT is the process by which epithelial cells are transformed into the mesenchymal cell phenotype, in which epithelial cells lose their original properties, namely contact adhesion and cusp polarity, and acquire mesenchymal cell characteristics of invasion, migration, and ECM-producing properties [12]. Myofibroblast transition and ECM overproduction are known to be induced by several profibrotic mediators, including transforming growth factor-β1 (TGFβ1), which is the most critical profibrotic factor, and its excessive secretion can cause lung fibrosis [13,14,15]. It is also considered the most important cytokine for the formation of lung fibrosis [16], and it can regulate various cell functions, such as proliferation, differentiation, migration, aging, and apoptosis [17,18]. TGF-β promotes fibrosis by inducing EMT processes in lung epithelial cells to become mesenchymal cells with a myofibroblast phenotype [19,20,21,22,23]. The downstream activity of TGF-β1 is mainly dependent on the Smad signaling pathway [24]. TGF-β1 induces phosphorylation of Smad2/3 protein, then forms a complex with Smad4, and ultimately the protein complex shifts to the nucleus to regulate the expression of target genes, which in turn trigger the EMT process in epithelial cells [25,26,27]. Therefore, blocking the TGF-β/Smad signaling pathway is considered a strategy to alleviate pulmonary fibrosis.

3-Cyclopropylmethoxy-4-(difluoromethoxy) benzoic acid (DGM) is an intermediate metabolite of an active small molecule compound that was discovered by chance in our laboratory while exploring active compounds in COPD, and with keen interest, we explored its activity in other diseases, including pulmonary fibrosis. Then we screened different molecules for their antifibrotic properties using TGF-β1 induced EMT cells. We determined that DGM showed the greatest potential to inhibit EMT in A549 cells. Therefore, in the present study, we evaluated the inhibitory effects of DGM on pulmonary fibrosis and its mechanism of action using in vivo and in vitro models. Altogether, the results indicated that DGM could be a potential candidate for the treatment of IPF.

## 2. Results

### 2.1. Effects of DGM on TGF-β1 Induced Viability of A549 and H1299 Cells

**Figure 1:**
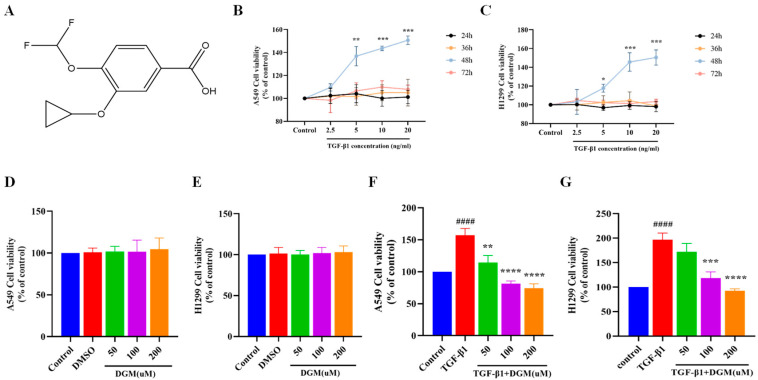
Effects of DGM on TGF-β1 induced proliferation of A549 and H1299 cells. Cells were treated with TGF-β1 and different concentrations (50, 100, and 200 µM) of DGM for 48 h. (**A**) Chemical structure of DGM. (**B**) A549 cell viability was evaluated using the MTT assay after stimulation of TGF-β1 at different concentrations for 24 h, 36 h, 48 h, and 72 h. (**C**) H12999 cell viability was evaluated by the MTT assay after stimulation of TGF-β1 at different concentrations for 24 h, 36 h, 48 h, and 72 h. (**D**) The cell viability of A549 cells and (**E**) H1299 cells treated with DGM (50, 100, 200 μM) for 24 h was determined by MTT assay. (**F**) A549 cells and (**G**) H1299 cells were treated with TGF-β1 and different concentrations of DGM (50, 100, and 200 µM) for 48 h. Values are presented as mean ± SEM (*n* = 6). * *p* < 0.05, ** *p* < 0.01, *** *p* < 0.001, **** *p* < 0.0001 vs. control group. #### *p* < 0.0001 vs. Control group; * *p* < 0.05, ** *p* < 0.01, *** *p* < 0.001, **** *p* < 0.0001 vs. TGF-β1 group.

To simulate the process of EMT of lung epithelial cells in vivo, as shown in Figure 1B,C, we selected two types of lung epithelial cells, A549 and H1299, and treated them with 2.5–20 ng/mL TGF-β1 for 24 h, 36 h, 48 h, and 72 h. We found that after 24 h, 36 h, and 72 h of stimulation, the two types of cells did not proliferate, but when TGF-β1 of various concentrations was used after 48 h of stimulation, the two types of cells could proliferate in a concentration-dependent manner; thus, 5 ng/mL TGF-β1 was selected for subsequent experiments. The incubation condition at 48 h mimics the process of EMT in vivo. To detect the cytotoxicity of DGM (Figure 1A) for subsequent experiments, we treated two types of epithelial cells with 50–200 μM DGM, as shown in Figure 1D,E. The MTT assay was used to detect the concentration of DGM within 200 μM, which had no toxic effect on the two cell lines after 48 h of treatment. Next, both cell lines were treated with TGF-β1 (5 ng/mL) for 48 h in combination with DGM. We found that DGM was effective in inhibiting TGF-β1 induced cell proliferation (Figure 1F,G). These results indicated that DGM did not exhibit toxicity in the two types of epithelial cells within our experimental dose range and also that DGM could inhibit the proliferation of epithelial cells stimulated by TGF-β1.

### 2.2. DGM Alleviated the EMT Process Induced by TGF-β1 in A549 Cells

**Figure 2:**
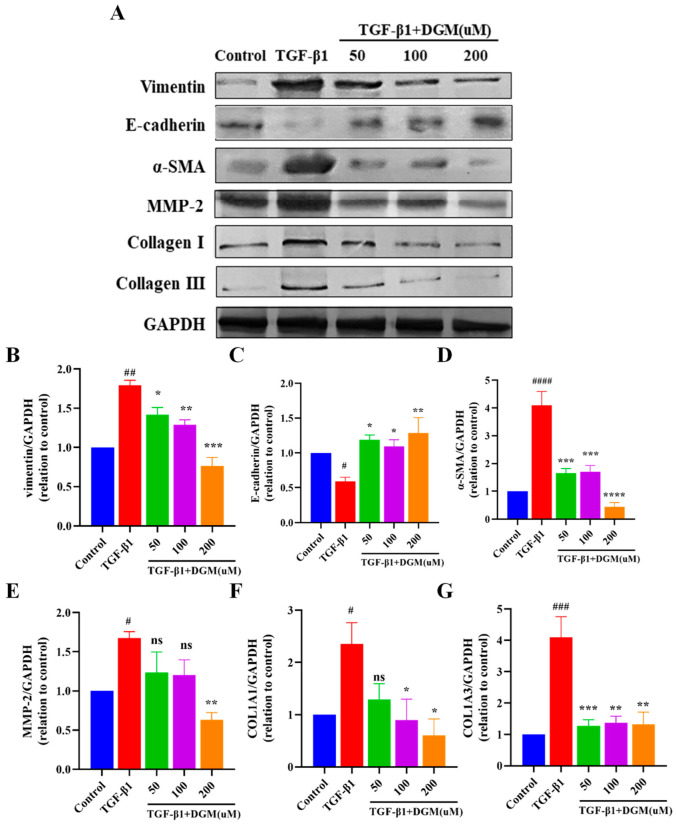
DGM alleviates the EMT process induced by TGF-β1 in A549 cells. A549 cells were treated with TGF-β1 in combination with different concentrations (50, 100, and 200 µM) of DGM for 48 h, and intracellular proteins were extracted for subsequent Western blot analysis. (**A**) Protein expression levels of vimentin, E-cadherin, α-SMA, MMP-2, Collagen Ⅰ, and Collagen III were evaluated by Western blotting. (**B**) Quantification of the vimentin/GAPDH ratio. (**C**) Quantification of the E-cadherin/GAPDH ratio. (**D**) Quantification of the α-SMA/GAPDH ratio. (**E**) Quantification of the MMP-2/GAPDH ratio. (**F**) Quantification of the Collagen Ⅰ/GAPDH ratio. (**G**) Quantification of the Collagen III/GAPDH ratio. Data represent the mean ± SEM (*n* = 3). # *p* < 0.05, ## *p* < 0.01, ### *p* < 0.001, #### *p* < 0.0001 vs. control group; * *p* < 0.05, ** *p* < 0.01, *** *p* < 0.001, **** *p* < 0.0001 vs. TGF-β1 group.

TGF-β1 plays a very important role in the EMT of lung epithelial cells. Thus, we treated A549 cells with TGF-β1 in combination with DGM to determine their effects on the EMT process (Figure 2A). As shown in Figure 2B–G, when A549 cells were stimulated with 5 ng/mL TGF-β1 for 48 h, vimentin, α-SMA, MMP-2, Collagen I, and Collagen III protein expression increased significantly, and E-cadherin protein expression was reduced. Instead, in the presence of DGM at a concentration of 200 μM, protein expression of vimentin, α-SMA, MMP-2, Collagen I, and Collagen III markedly decreased, and E-cadherin protein expression increased.

### 2.3. DGM Inhibited the Activation of the Smad Signaling Pathway Stimulated by TGF-β1

**Figure 3:**
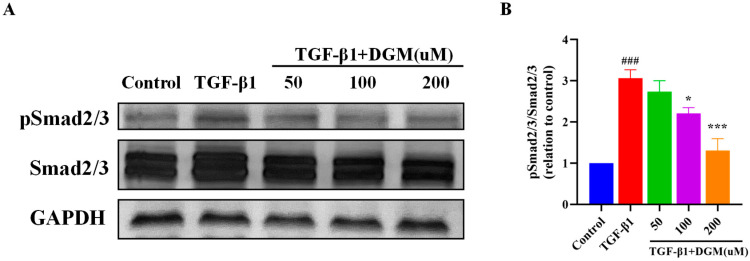
DGM inhibits the activation of the Smad signaling pathway stimulated by TGF-β1. A549 cells were pretreated with DGM for 2 h, followed by TGF-β1 (5 ng/mL) stimulation for an additional 6 h, and intracellular proteins were extracted for subsequent western blotting analysis. (**A**) Western blotting analyses of Smad2/3, pSmad2/3 protein expression in TGF-β1-stimulated A549 cells stimulated with TGF-1. (**B**) Quantification of the pSmad2/3/Smad2/3 ratio. Data represent the mean ± SEM (*n* = 3). ### *p* < 0.001 vs. control group; * *p* < 0.05, *** *p* < 0.001 vs. TGF-β1 group.

To further investigate the mechanism of inhibition by DGM of TGF-β1 induced EMT of A549, we investigated the effects of DGM on activation of the Smad signaling pathway. As shown in Figure 3A, after stimulation of A549 cells with TGF-β1, the protein expression of Smad 2/3 changed hardly, and the protein level of phosphorylated Smad2/3 was obviously increased. After DGM treatment, the expression of phosphorylated Smad2/3 decreased markedly, especially at the concentration of 200 μM. The ratio of p-Smad2/3 to Smad2/3 for different groups is shown in Figure 3B. The above results suggest that DGM inhibition of EMT-related protein expression could be achieved by inhibiting activation of the Smad signaling pathway.

### 2.4. DGM Improved Bleomycin-Induced Weight Loss and Decreased Lung Function in Rats

**Figure 4:**
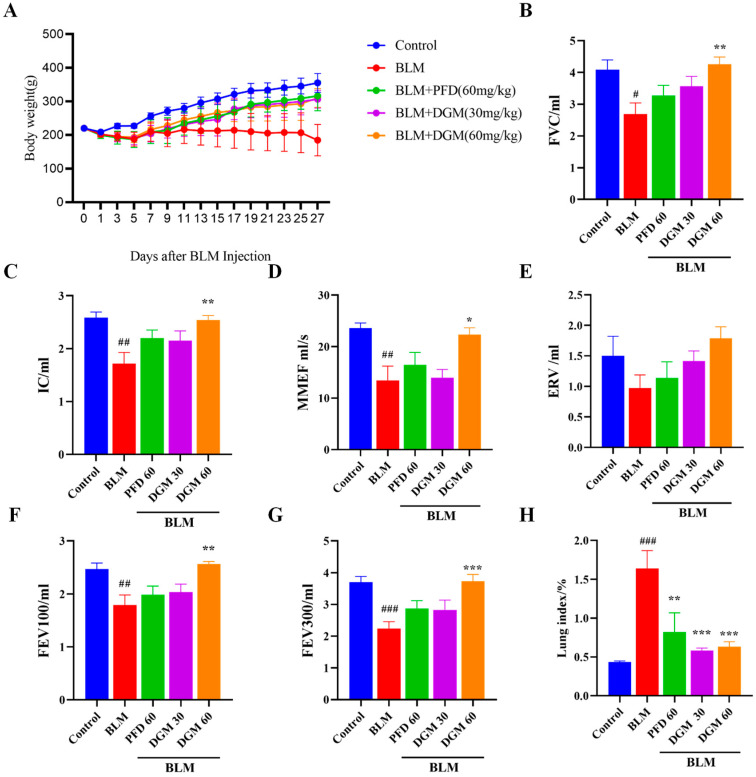
DGM improves BLM-induced weight loss and decreases lung function in rats. (**A**) Body weight records of rats in the control group, the bleomycin (BLM) group, the PFD group, the DGM (30 mg/kg), and DGM (60 mg/kg) groups throughout the experiment. (**B**–**G**) Pulmonary function indicators: forced vital capacity (FVC), deep inspiratory volume (IC), maximum mid-expiratory flow (MMEF), supplementary expiratory volume (ERV), forced expiratory volume (FEV). (**H**) Lung coefficient: lung weight/body weight. Data represent the mean ± SEM (*n* = 6). # *p* < 0.05, ## *p* < 0.01, ### *p* < 0.001 vs. control group; * *p* < 0.05, ** *p* < 0.01, *** *p* < 0.001 vs. BLM group.

To study the anti-IPF activity of DGM in vivo, we treated rats with bleomycin (BLM) to establish a model of pulmonary fibrosis. As shown in Figure 4A, BLM could lead to a continuous loss of body weight in the first 7 days when compared to the control group. Compared to the BLM-induced group, the weight of rats in the DGM treatment groups improved markedly. Next, we used lung function indicators (PFT Animal Lung Function Test System) to assess the severity of pulmonary fibrosis in each treatment group. Forced vital capacity (FVC), deep inspiratory volume (IC), forced expiratory volume (FEV), maximum mid-expiratory flow (MMEF), supplementary expiratory volume (ERV), and other indices in the BLM group were significantly lower than in the control group, while lung function was significantly improved by DGM administration. The DGM-treated group showed a stronger effect than PFD treatment at the same dose, with 60 mg/kg DGM achieving a greater effect than 30 mg/kg DGM (Figure 4B–G). When comparing the control group with the BLM-induced group, the lung coefficient of the model group was much higher than that of the control group, and after DGM treatment, the lung coefficient decreased significantly, with the high-dose treatment achieving a better effect than that of lower doses (Figure 4H).

### 2.5. DGM Reduced BLM-Induced Lung Inflammation and Fibrosis

**Figure 5:**
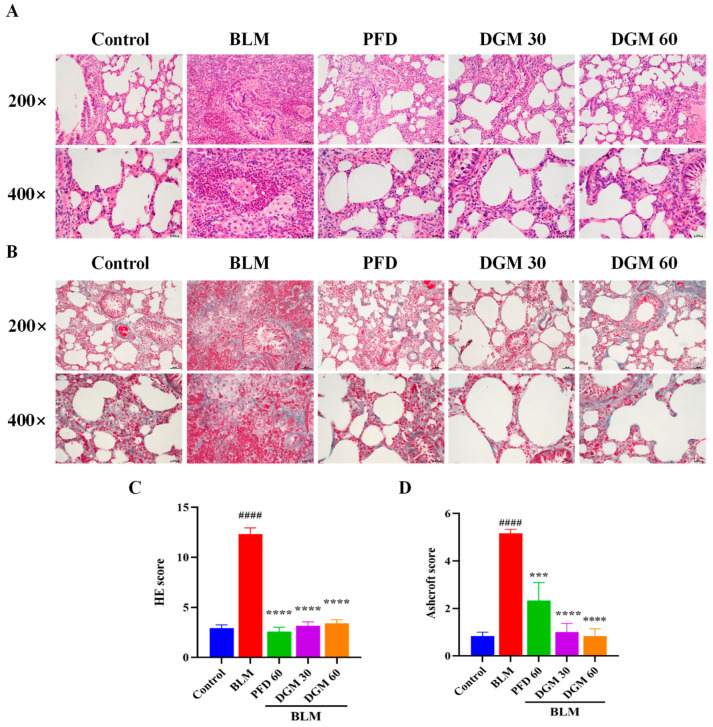
DGM reduces BLM-induced lung inflammation and fibrosis. (**A**) Sections of lung tissue stained with H&E. (**B**) Masson’s trichrome staining of paraffin sections from different experimental groups of rats. (**C**) Bar graph showing the scale score for all groups of H&E. (**D**) Bar graph showing the Ashcroft scale score for all Masson groups. Data represent the mean ± SEM (*n* = 6). #### *p* < 0.0001 vs. control group; *** *p* < 0.001, **** *p* < 0.0001 vs. BLM group.

To investigate the effects of DGM on lung fibrosis in rats, we used a BLM-induced rat model of pulmonary fibrosis. On Day 0, the BLM group, the DGM group, and the PFD group were subjected to a tracheal drip BLM solution, and animals were euthanized around Day 28. During this period, the control group and the BLM group were injected with normal saline, and the administration group was injected with different drugs for treatment. As shown in Figure 5A, the lung tissue of the control group by H&E staining exhibited an intact alveolar wall structure, an intact bronchial structure, and no shedding or hyperplasia of the bronchial epithelial cells, while the alveolar wall structure of the lung tissue of rats in the BLM group was destroyed, pulmonary consolidation occurred, bronchial epithelial cells exhibited sloughing and hyperplasia, and secretions could be seen in the lumen. The lung consolidation of the PFD group was significantly reduced, although pathological changes, such as thickening of the alveolar wall, hyperemia and edema of lung tissue, and cell infiltration of pulmonary interstitial cells, could still be observed. Lung inflammation was obviously reduced in the DGM group, and the pathological score of the high-dose DGM group had a better effect than that of the low-dose group. Masson staining was used to evaluate the fibrosis and collagen deposition of rat lung tissue. Compared to the control group, the area of lung tissue fibrosis of the BLM group was significantly increased, the thickness of the alveolar membrane was significantly thicker, and the lung tissue was significantly destroyed. Compared to the BLM group, the degree of collagen deposition and fibrosis in the DGM group and the PFD group improved significantly, collagen deposition in the low-dose DGM group was less than that of the PFD group, and the effect of the high-dose DGM group was better than that of the low-dose group. These results suggest that DGM reduces the degree of lung damage and fibrosis caused by BLM at doses of 30 mg/kg and 60 mg/kg (Figure 5B).

### 2.6. DGM Reduced the Expression of α-SMA, Hydroxyproline, and Total Collagen in Lung Tissue

**Figure 6:**
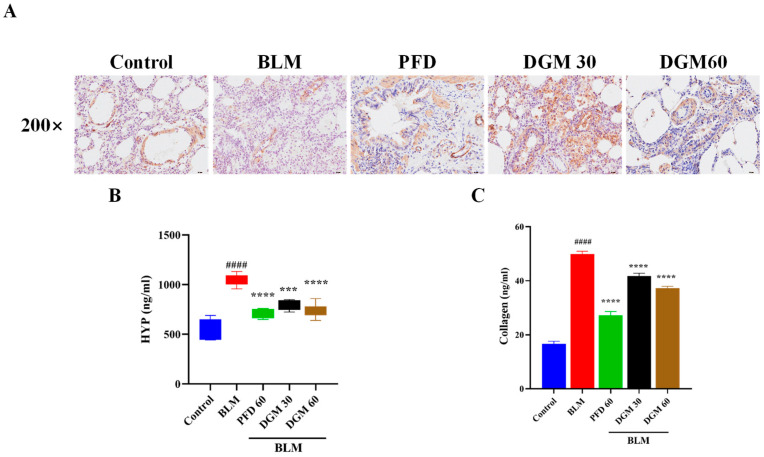
DGM reduces the expression of α-SMA, hydroxyproline, and total collagen in lung tissue. (**A**) Expression of α-SMA was investigated by immunohistochemical staining. (**B**) HYP levels were examined by ELISA. (**C**) Collagen levels were examined by ELISA. Data represent the mean ± SEM (*n* = 6). #### *p* < 0.0001 vs. control group; *** *p* < 0.001, **** *p* < 0.0001 vs. BLM group.

In the pulmonary fibrosis process, fibroblast differentiation into myofibroblasts is a very critical stage, and the expression of α-SMA was characteristic of myofibroblasts. Hydroxyproline is a main component of collagen, and overexpression of collagen is also considered a characteristic of the ECM. Next, the expression of α-SMA, HYP, and total collagen in lung tissue was detected by immunohistochemical staining and enzyme-linked immunosorbent assays. As shown in Figure 6A, compared to the control group, the expression of α-SMA in the BLM group increased significantly, and its expression was significantly reduced after DGM and PFD treatment. As shown in Figure 6B,C, the expression of HYP and total collagen increased significantly in the BLM group, and treatment with DGM (30 mg/kg and 60 mg/kg) treatment significantly improved their expression.

### 2.7. DGM Reduced the Content of Inflammatory Cells and Inflammatory Cytokines in Rat Bronchoalveolar Lavage Fluid

**Figure 7:**
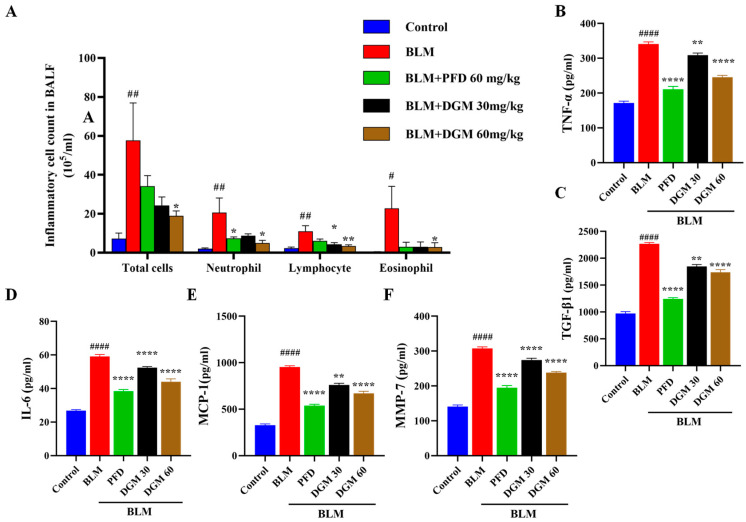
DGM can reduce the content of inflammatory cells and inflammatory cytokines in the rat BALF. (**A**) Twenty-four hours after the last administration, total inflammatory cells, neutrophils, lymphocytes, and eosinophils were counted in the BALF. (**B**) TNF-α, (**C**) TGF-β1, (**D**) IL-6, (**E**) MCP-1, and (**F**) MMP-7 levels of the BALF. Data represent the mean ± SEM (*n* = 6). # *p* < 0.05, ## *p* < 0.01, #### *p* < 0.0001 vs. control group; * *p* < 0.05, ** *p* < 0.01, **** *p* < 0.0001 vs. BLM group.

We examined the effects of DGM on the inflammatory cell infiltration in the bronchoalveolar lavage fluid (BALF) of rats. As shown in Figure 7A, compared to the control group, the total number of inflammatory cells, neutrophils, lymphocytes, and eosinophils in the BALF of rats in the BLM group increased significantly. At the same time, compared to rats in the BLM group, DGM administration significantly reduced the number of total inflammatory cells, neutrophils, lymphocytes, and eosinophils in BALF, and the effect of 60 mg/kg was better than 30 mg/kg treatment. 

To assess whether DGM influenced TNF-α, TGF-β1, IL-6, and other fibrotic factors in the fibrosis process of rats, we used the ELISA assay to detect cytokine levels in the BALF of rats. Compared to the control group, BLM significantly increased the production of TNF-α, TGF-β1, IL-6, MCP-1, and MMP-7 production. Compared to the BLM group, DGM administration inhibited the increase of TNF-α, TGF-β1, IL-6, MCP-1, and MMP-7 in a dose-dependent manner (Figure 7B–F). These results showed that BLM induced the accumulation of inflammatory cells in rat lung tissue and secreted several cytokines to accelerate the fibrosis process, while the intervention with DGM reduced the number of inflammatory cells in the lungs of rats and attenuated the secretion of these cytokines.

### 2.8. DGM Repressed the BLM-Induced EMT and Fibroblast Activation in Rat Lung Tissue

**Figure 8:**
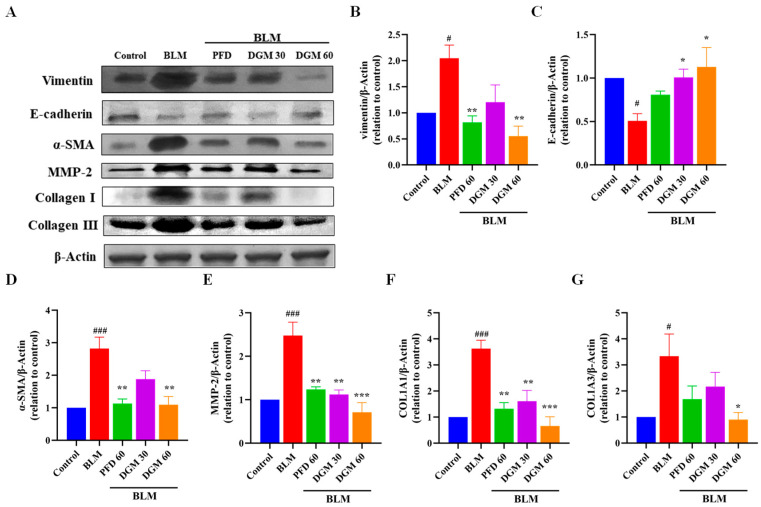
DGM repressed the bleomycin-induced EMT and fibroblast activation in rat lung tissue. (**A**) Western blotting analyses of vimentin, E-cadherin, α-SMA, MMP-2, Collagen I, and Collagen III protein expression in lung tissues. (**B**) Quantification of the vimentin/β-actin ratio, (**C**) E-cadherin/β-actin ratio, (**D**) α-SMA/β-actin ratio, (**E**) MMP-2/β-actin ratio, (**F**) Collagen Ⅰ/β-actin ratio, and (**G**) Collagen III/β-actin ratio. Data represent the mean ± SEM (*n* = 6). # *p* < 0.05, ### *p* < 0.001, vs. control group; * *p* < 0.05, ** *p* < 0.01, *** *p* < 0.001, vs. BLM group.

To determine whether DGM could alleviate BLM-induced pulmonary fibrosis, the expression of the EMT marker proteins and fibroblast activation marker protein: vimentin, E-cadherin, α-SMA and collagen I, collagen III, as well as the enzyme MMP-2 related to the degradation of the ECM of lung tissue, were detected. As shown in Figure 8A, the expression of lung tissue vimentin, α-SMA, collagen I, collagen III, and MMP-2 was significantly elevated, and the expression of E-cadherin was significantly reduced compared to the control group. The DGM 30 mg/kg and 60 mg/kg groups were able to significantly down-regulate the expression of the vimentin, α-SMA, collagen I, collagen III, and MMP-2 proteins in lung tissue and up-regulate the expression of E-cadherin. Statistical analysis of protein levels showed that DGM could inhibit vimentin, α-SMA, collagen I, collagen III, and MMP-2 expression and showed a dose-dependent relationship and higher E-cadherin expression in a dose-dependent manner (Figure 8B–G). The findings showed that DGM could inhibit the EMT of rat lung tissue induced by BLM, thus inhibiting the fibroblast activation to alleviate pulmonary fibrosis.

## 3. Discussion

Alveolar epithelial cell injury and subsequent repair are the main factors involved in the irreversible pathogenesis of IPF [28,29]. Alveolar epithelial cells are the main players in the composition of alveolar structures and play a critical role in lung tissue homeostasis [30]. Type II alveolar epithelial cells are the most important cells on the alveolar surface, and when stimulated by environmental pathogens, in order to protect the alveolar surface, type II alveolar epithelial cells repair epithelial tissue through proliferation and differentiation [31]. In this study, DGM inhibited cell proliferation induced by TGF-β1 in A549 cells and H1299 cells, suggesting that DGM could slow the development of fibrosis by improving the proliferation of type II alveolar epithelial cells. To rule out that the inhibitory effects of DGM in the proliferation of type II alveolar epithelial cells induced by TGF-β1 may be related to toxicity, we evaluated cell viability using the MTT assay, and the results showed that DGM had no significant toxic effect on both cell lines within the indicated concentration range. In this study, we demonstrated that DGM could inhibit TGF-β1-induced proliferation of A549 cells, suggesting that DGM could slow down the development of pulmonary fibrosis at some level by inhibiting the proliferation of type II epithelial cells in the presence of injury, thereby reducing the occurrence of the EMT process.

TGF-β1 is considered to be a cellular regulator of fibrosis in a variety of organs, especially in the lung, and has a certain regulatory effect on ECM protein deposition, inflammation, and tumorigenesis [32]. Under stimulation of TGF-β1, type II alveolar epithelial cells undergo EMT, epithelial cells lose epithelial cell characteristics, acquire the fibroblast phenotype, and develop fibroblast foci and cause excessive protein deposition in the ECM [33]. EMT is involved in the development of many diseases and plays a very important role in pulmonary fibrosis. On treating A549 cells with TGF-β1 in vitro to mimic the EMT process, we observed an up-regulation in the expression of vimentin, α-SMA and collagen, EMT-related biomarkers, and the main components of the ECM. Further, DGM could attenuate this up-regulation. E-cadherin, the most critical indicator of EMT, was down-regulated on exposure to TGF-β1 stimulation [34], and DGM attenuated this change. These results suggest that DGM inhibits excessive ECM deposition by inhibiting the occurrence of EMT. At the same time, we found that DGM could reduce the up-regulation of MMP-2 induced by TGF-β1, suggesting that this reversal effect by DGM was related to the degradation of ECM components. Fibroblasts are also very critical cells in the development and progression of lung fibrosis. In this study, we have mainly demonstrated that DGM could inhibit the occurrence of EMT in epithelial cells, but whether there is an effect on the activation of fibroblasts needs to be explored more. Many signaling pathways are involved in the biological processes of EMT, such as Wnt/β-catenin, NF-κB, and TGF-β1/Smad [12]. The TGF-β1/Smad signaling pathway is the most cited EMT-related signaling pathway. TGF-β1 binds to its receptor on the membrane, which subsequently leads to phosphorylation of Smad2/3, which in turn binds to Smad2/3 to form a heterologous complex, which translocates to the nucleus where it regulates the expression of target genes [35,36,37]. DGM decreased the phosphorylation levels of Smad2 and Smad3 stimulated by transforming growth factor-β1, suggesting that DGM has the ability to inhibit transforming growth factor-β1/Smad-dependent signaling pathways. DGM may inhibit the stimulation of the type II alveolar epithelial transformation process by inhibiting Smad2/3 phosphorylation and stimulating the EMT process of type II alveolar epithelial cells. The specific regulatory mechanism for inhibition needs to be further studied.

Pulmonary function testing (PFT) is an important clinical examination and diagnostic indicator of pulmonary fibrosis, and the lung function of patients with IPF will continue to decline. In this study, a PFT animal lung function assay was used to measure lung function. The results showed that DGM reversed the changes in various indicators of lung function in rat models of IPF, and the effect was better than that of the positive drug PFD at high doses. Combined with the comprehensive analysis of the above lung function indicators, our findings strongly suggest that DGM improved lung function of IPF rats.

Inflammation is observed throughout the course of pulmonary fibrosis. In the early stage of the BLM-induced pulmonary fibrosis model in rats, damage to alveolar epithelial cells led to a large release of crude pro-inflammatory fibrosis biomarkers and increased neutrophil infiltration, which caused a strong inflammatory response in the lungs. With the progression of the disease, chronic inflammation dominated by lymphocyte infiltration will occur in the later stage [38], therefore inhibiting the inflammatory response is conducive to blocking the further development of pulmonary fibrosis. The experimental results showed that after BLM stimulation, the overall number of inflammatory cells in the BALF increased significantly, especially neutrophils, lymphocytes, and eosinophils. DGM significantly and dose-dependently reduced the total number of inflammatory cells, with the 60 mg/kg dose being more effective than that of PFD treatment. TNF-α, TGF-β1, IL-6, MCP-1, and MMP-7 are inflammatory mediators and profibrotic factors involved in lung tissue damage and play a significant role in the initiation and progression of pulmonary fibrosis [39,40,41,42,43]. Therefore, we measured the levels of these cytokines in the BALF supernatant. The results showed that DGM could reduce the levels of inflammatory factors. Pathomorphological H&E staining also showed a decrease in structural alveolar lesions and a decrease in inflammatory cell infiltration. These results indicated that DGM could reduce lung inflammation and protect alveolar epithelial cells, which requires further experimental verification. The above results showed that DGM could slow the progression of pulmonary fibrosis by inhibiting lung inflammation.

Pulmonary fibroblast activation is an important factor in the development of pulmonary fibrosis, and fibroblasts are activated by processes such as EMT and endothelial–mesenchymal transformation, and a large amount of ECM is deposited in lung tissue, resulting in changes in lung structure [44]. Integrins activate downstream pathways to promote the transformation of fibroblasts into myofibroblasts [45]. Myofibroblasts produce large amounts of collagen, characterized by the presence of α-SMA stress fibers. The HYP content in lung tissue is often used as a fibrosis marker to characterize the degree of collagen deposition in fibrotic tissue. Immunohistochemistry, WB, and ELISA experiments have shown that DGM could reduce ECM deposition and reduce lung fibroblast activation, thus exerting antifibrotic effects. 

Previously, our laboratory studied the activity of the marine derivative MBH-212 in the treatment of COPD, and its intermediate metabolite, DGM, in pulmonary fibrosis. In summary, DGM inhibits the alveolar epithelial cell EMT process by inhibiting the TGF-β1/Smad signaling pathway in vitro, and slows the development of pulmonary fibrosis in vivo by reducing lung inflammation, improving lung function, and reducing remodeling of the ECM. However, the effects of DGM on non-Smad-dependent signaling pathways are unclear and require further study. This current study actually provides another piece of the puzzle on the overall picture of the benefits and effectiveness of DGM in acute and chronic lung disease, although the detailed mechanism of action urgently needs to be elucidated. This is the first study of the effect of DGM on pulmonary fibrosis, and this paper explores the selectivity of benzoic acid compounds in IPF, but it is unclear whether DGM can improve pulmonary fibrosis by modulating other factors, and further research is needed. Whether the same therapeutic effect is achieved when administered after 14 days of tracheal drip requires further study.

## 4. Materials and Methods

### 4.1. Drugs and Reagents

3-Cyclopropylmethoxy-4-(difluoromethoxy) benzoic acid and PFD were purchased from Aladdin Reagent Co., Ltd. (Shanghai, China). BLM was purchased from Yuanye Biotechnology Co., Ltd. (Shanghai, China). TGF-β1 was purchased from PEPROTECH (Cranbury, NJ, USA). Rats TNF-α, TGF-β1, IL-6, MCP-1, MMP-7, HYP, and collagen enzyme-linked immunosorbent assay (ELISA) kits were purchased from Shanghai FANKEW Industrial Co., Ltd. (Shanghai, China). The BCA protein detection kit was purchased from Beyotime Biotechnology (Shanghai, China). Antibodies against vimentin (5741S), E-cadherin (3195S), α-SMA (19245S), MMP-2 (40994S), Collagen I (72026T), Smad2/3 (8685S), and P-Smad2/3 (8828S) were obtained from Cell Signaling Technology (CST; Beverly, MA, USA). An antibody against collagen III (ab7778) was obtained from Abcam (Cambridge, UK). Antibody β-actin (GB11001) and GAPDH (GB11002) were purchased from Sewell Biotechnology Co., Ltd. (Wuhan, China).

### 4.2. Cell Culture and Treatment

The A549 cell line was purchased from the Chinese Academy of Sciences Cell Bank (Shanghai, China) and cultured in RPMI.1640 culture medium with 10% inactivated fetal bovine serum in an incubator with 5% CO_2_ at 37 °C. A549 cells were seeded in a 96-well plate for 12 h, then underwent serum starvation for 12 h. Treatment with 5 ng/mL TGF-β1 for 48 h was used to induce the in vitro EMT model.

### 4.3. MTT Assay

After A549 cells were treated with different concentrations (50, 100, 200 μM) of the test drug for 48 h, cell viability was detected by the MTT assay. A 5 mg/mL MTT solution was added to a 96-well plate treated with drugs, incubated in a 37 °C incubator for 4 h, then dissolved with DMSO to form crystals. Finally, absorbance was measured at 490 nm with a microplate reader.

### 4.4. Western Blotting Analysis

A549 cells were seeded in 6-well plates at a density of 5 × 10^5^, cultured for 12 h, serum starved for 12 h, and then stimulated with TGF-β1 for 24 h. Cells were then lysed to extract protein by adding a 150 μL volume of lysis buffer (containing protease inhibitors) to each well of the 6-well plate. Cells were lysed on ice for 25 min, and lysates were collected in a centrifuge tube by cell scraping. The lysate was centrifuged at 14,000 rpm at 4 °C for 5 min, the supernatant was removed, and the pellet was discarded. The protein concentration of the extracted supernatant was measured using the BCA assay. For electrophoresis, 25 g of protein was added to each well, and different separation gels were selected according to the different molecular weights of the target protein. Proteins were then transferred to NC membrane, blocked with 5% BSA for 2 h, and then exposed to the corresponding primary antibody and incubated overnight at 4 °C. The next day, the primary antibody solution was recovered. The film was washed 6 times with 1 × TBST for 6 min each time. The corresponding secondary antibody solution was added after washing and incubated at room temperature for 1 h. The film was washed 6 times, and then the strips were visualized with the ECL luminescence kit. Finally, ImageJ software was used for grayscale densitometric analysis.

### 4.5. Animal Models

Male Sprague–Dawley rats (aged 6–8 weeks) were obtained from Jinan Pengyue Laboratory Animal Co., Ltd. (Jinan, China). The living environment of the rats provided adequate water and food, an ambient temperature of 25 °C, and a light/dark cycle of 12 h. Before the beginning of the experiment, rats were placed in an experimental environment and reared adaptively for one week. All animal experiments were conducted according to the guidelines of the Animal Experiment Ethics Committee of Ocean University of China (OUC-SMP-2022-06-01).

### 4.6. Mold Making and Treatment Scheme

The 60 rats were randomly divided into five groups of 12 rats, namely: (1) control group, (2) BLM group, (3) BLM + PFD (60 mg/kg) group, (4) BLM + DGM (30 mg/kg) group, and the (5) BLM + DGM (60 mg/kg) group. PFD was used as the positive test drug. Prior to tracheal drip, all rats were injected with 4% chloral hydrate solution for anesthesia. To establish the pulmonary fibrosis (PF) model, rats received BLM (5 mg/kg, volume 100 µL/200 g of body weight) dissolved in sterile PBS by intratracheal instillation. Subsequently, the BLM + PFD group, BLM + DGM (30 mg/kg) group, and BLM + DGM (60 mg/kg) group were treated every two days; the BLM group and the control group were given the same amount of normal saline, and the rats were euthanized on Day 28 and sampled for testing.

### 4.7. Pulmonary Function Tests

The rats were anesthetized by injection of 4% chloral hydrate, cutting the skin at the midline of the neck, bluntly peeling off the exposed trachea, cutting a small opening in the trachea with small scissors, inserting the head of the pulmonary function tester cannula into the trachea, and starting running a PFT Animal Lung Function Test System, which was purchased from Shanghai Tawang Intelligent Technology Co. (Shanghai, China),to test the lung function of the rats.

### 4.8. Lung Coefficient Detection

After the rat was euthanized, lung tissue was sampled. All residual blood stains on the tissue were washed with PBS. Excess connective tissue was removed, and then the water was removed with filter paper. The lung tissue was weighed, and the weight was divided by the weight of the rat before being euthanized and then multiplied by 100%, which defined the lung coefficient.

### 4.9. H&E and Masson’s Staining

After sacrifice of the rat, the lung tissue was removed and immersed in 4% paraformaldehyde. It was then dehydrated, embedded, sliced, and stained with H&E and Masson, respectively. Finally, pathological changes such as lung tissue inflammatory infiltration and collagen deposition were observed under a light microscope, and three random fields were randomly selected for each sample and taken at magnifications of 200× and 400×.

### 4.10. Immunohistochemical Staining

Detection of α-SMA expression in rat lung tissue by immunohistochemistry. Briefly, tissue sections were pretreated in a microwave oven to inactivate enzymes, blocked, and reacted with primary and secondary antibodies. The samples were chromogenized, counterstained, decolorized, sealed, and finally scanned with a digital pathology slide scanner.

### 4.11. Determination of Hydroproline and Collagen

Rat lung tissue was stored at −80 °C and once thawed, RIPA lysis buffer was added, placed in a Xianou-24 homogenizer for homogenization, and allocated to the corresponding wells of 96-well culture plates, as determined by ELISA kits, which were purchased from FANKEI Industrial Co., Ltd. (Shanghai, China).

### 4.12. BALF Collection and Inflammatory Cell Count

After anesthetizing the rat, the trachea was exposed, and the rat was then intubated. The lung tissue was rinsed twice with 1.5 mL of pre-cooled PBS, and BALF was collected in a 4 mL centrifuge tube, followed by centrifugation at 2000 rpm for 10 min. The supernatant was collected and stored at −80 °C for subsequent cytokine detection. The BALF precipitate was suspended in 200 μL of PBS, and the total numbers of white blood cells, neutrophils, lymphocytes, and eosinophils in the BALF were determined with ProCyte Dx^®^ Hematology Analyzer (IDEXX). Laboratories Inc., Westbrook, ME, USA).

### 4.13. ELISA Method for the Detection of Cytokines in BALF Supernatant

The levels of TGF-β1, TNF-α, IL-6, MCP-1, and MMP-7 in the BALF were determined by ELISA, as indicated by the corresponding kit instructions.

### 4.14. Statistical Analysis

Data are expressed as the mean ± SEM. GraphPad Prism 9.3.1 software (San Diego, CA, USA) was used for statistical analysis, and one-way analysis of variance (ANOVA) followed by multiple comparison tests was performed. Statistical significance was accepted at *p* < 0.05.

## Data Availability

Not applicable.

## Abbreviations

MTT	3-(4,5-dimethylthiazol-2-yl)-2,5-diphenyl tetrazolium bromide
FBS	fetal bovine serum
BLM	bleomycin
PFD	pirfenidone
α-SMA	α-smooth muscle actin
HYP	hydroxyproline
BALF	bronchoalveolar lavage fluid
MMP-7	matrix metallopeptidase 7
